# Wild-Type Transthyretin Cardiac Amyloidosis in a Transplanted Heart

**DOI:** 10.1016/j.jaccas.2023.101935

**Published:** 2023-06-23

**Authors:** Lily K. Stern, Pamela A. Ivey, Corey J. Lum, Shayaan Zaidi, Daniel Luthringer, Angela Velleca, Jon A. Kobashigawa, Jignesh K. Patel, Michelle M. Kittleson

**Affiliations:** a Department of Cardiology, Smidt Heart Institute, Cedars-Sinai Medical Center, Los Angeles, California, USA; b Department of Cardiology, Intermountain Healthcare, Las Vegas, Nevada, USA; c Department of Pathology, Cedars-Sinai Medical Center, Los Angeles, California, USA

**Keywords:** amyloid cardiomyopathy, cardiac amyloidosis, heart transplantation, wild-type transthyretin

## Abstract

Wild-type transthyretin amyloid cardiomyopathy (ATTRwt-CM) is more prevalent than appreciated in the elderly. We present the case of an 88-year-old woman who underwent heart transplantation for ischemic cardiomyopathy and then presented 21 years later with new onset atrial flutter, found on endomyocardial biopsy to have new ATTRwt-CM. (**Level of Difficulty: Advanced.**)

## Central Illustration

**Figure:**
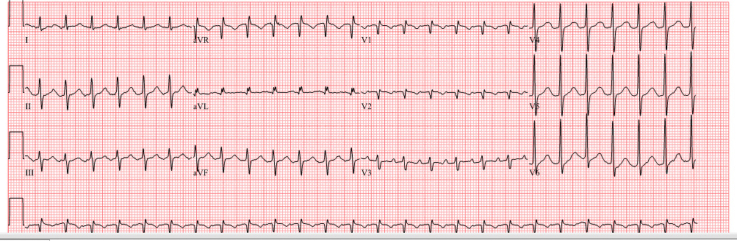

**Figure 1:**
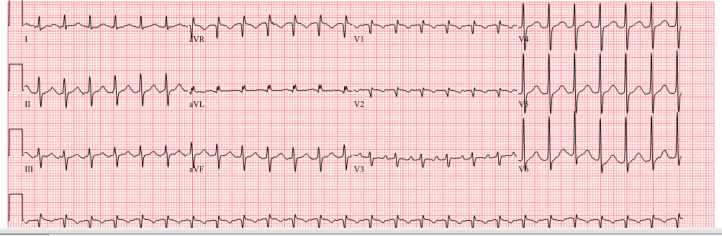
Electrocardiogram Showing Atrial Flutter With 2:1 Ventricular Conduction

An 88-year-old woman presented to the emergency department after a mechanical fall. The patient’s heart rate was 150 beats/min, blood pressure was 147/92 mm Hg, and oxygen saturation was 98% on room air. Her examination results were notable for a regular tachycardia and mild confusion. Laboratory studies revealed a B-type natriuretic peptide level of 962 ng/L and troponin of 9.8 ng/L (normal range 0-53.7 ng/L). Chemistry studies revealed potassium 3.7 mmol/L, magnesium 2.1 mg/dL, creatinine 1.4 mg/dL, and glucose 110 mg/dL. An electrocardiogram showed atrial flutter with 2:1 ventricular conduction (Figure 1). An echocardiogram demonstrated left ventricular ejection fraction 40% to 45% (65% 3 months prior) (Videos 1 and 2).

**Learning Objectives**

• To understand risk factors for development of rejection and cardiac allograft vasculopathy in patients with remote heart transplantation.
• To identify the presentation and natural history of wild-type transthyretin amyloid cardiomyopathy.
• To consider non–transplant-related cardiac conditions in patients as they live longer after heart transplantation.

## Medical History

The patient had ischemic cardiomyopathy, for which she had undergone heart transplantation (HTx) 21 years prior. She had an ischemic stroke of a presumed atherosclerotic source 6 years before this presentation, without residual deficits; she also had shoulder and hip arthropathies and recently diagnosed dementia with missed immunosuppression doses resulting in subtherapeutic tacrolimus levels.

## Differential Diagnosis

Atrial tachyarrhythmia and newly reduced left ventricular systolic function after HTx raised a concern for rejection or cardiac allograft vasculopathy (CAV).

Although acute rejection is rare >20 years after HTx,^1^ given the patient’s missed immunosuppression doses with subtherapeutic levels, this diagnosis was possible. A more common cause of late graft dysfunction is CAV,^1^ which is characterized by: 1) intimal hyperplasia of the epicardial coronary arteries, which may result in ischemia from coronary stenoses; and 2) microvascular disease, which may lead to fibrous scar tissue and restrictive physiology. Risk factors for CAV include prior allograft rejection, anti-HLA antibody sensitization, cytomegalovirus infection, ischemia-reperfusion injury at the time of HTx, and traditional risk factors for coronary artery disease.^2^

## Investigations

**Figure 2:**
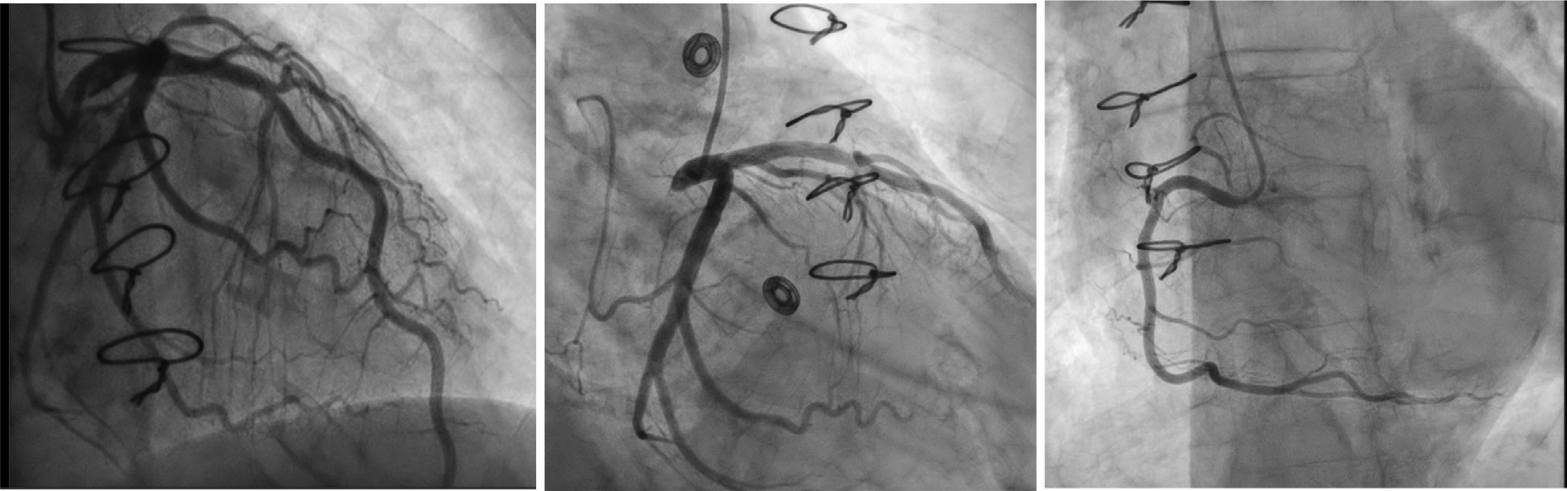
Coronary Angiogram Showing Normal Epicardial Coronary Arteries

A coronary angiogram revealed normal epicardial coronary arteries (Figure 2). Right heart catheterization revealed right atrial pressure of 3 mm Hg, pulmonary artery pressure of 21/8 (mean 15) mm Hg, and mean pulmonary capillary wedge pressure of 8 mm Hg. Cardiac index was 1.51 and 2.19 L/min/m^2^ by thermodilution and Fick method, respectively.

**Figure 3:**
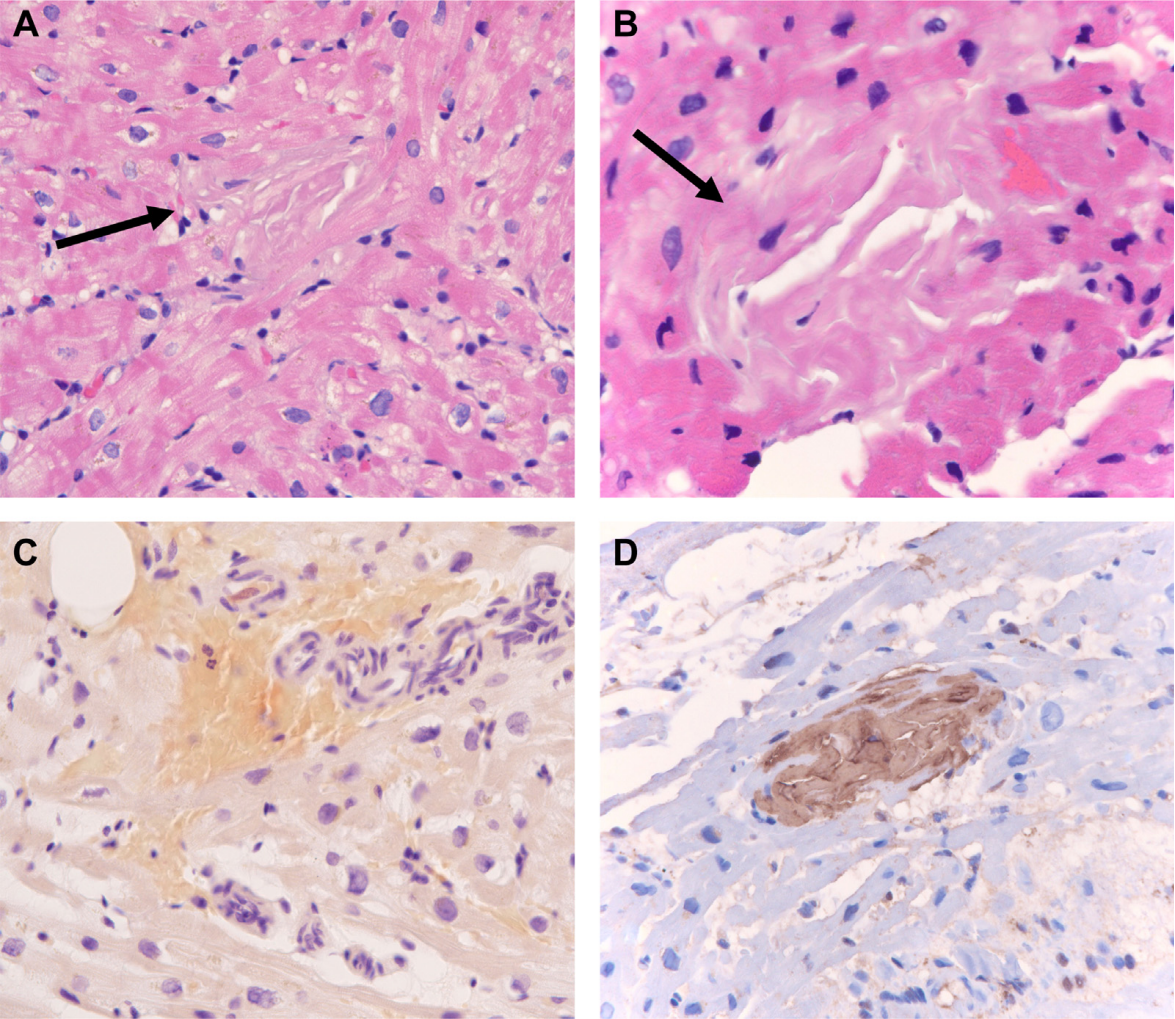
Endomyocardial Biopsy Specimens Demonstrating Transthyretin Amyloid Photomicrographs of hematoxylin and eosin-stained sections of myocardium **(A, B)** showing amyloid deposits **(arrows)** characterized by acellular deposits of pink extracellular matrix material that are devoid of cells and exhibit cracking. These deposits stain light red/orange by Congo Red stain **(C)**. Immunohistochemical staining (brown chromogen) shows intense reactivity with antibodies against transthyretin **(D)**.

Three endomyocardial biopsy (EMBx) samples showed no cellular or antibody-mediated rejection. However, histopathologic evaluation revealed acellular eosinophilic extracellular matrix material deposits, weakly positive on Congo red staining with apple-green birefringence on polarization microscopy indicative of cardiac amyloidosis. Secondary immunohistochemical testing demonstrated reactivity of these deposits with antibodies against transthyretin (TTR) (Figure 3). Liquid chromatography/tandem mass spectrometry confirmed the presence of transthyretin type amyloid and further showed no abnormality in the amino acid sequence of the transthyretin gene (*TTR*), consistent with wild-type transthyretin amyloid cardiomyopathy (ATTRwt-CM). The original heart explant from 21 years prior was reviewed again and confirmed to have no amyloid deposits.

## Management

On admission to the hospital, the patient was empirically treated for acute allograft rejection with high-dose intravenous methylprednisolone 500 mg for 3 days while EMBx results were awaited. She received metoprolol succinate 25 mg twice daily and intravenous heparin. The following day, she experienced spontaneous conversion to normal sinus rhythm and received apixaban 5 mg twice daily at discharge. She was prescribed tafamidis and continued her preadmission immunosuppression maintenance regimen.

## Discussion

Cardiac amyloidosis is a multisystem condition in which normally soluble proteins misfold and aggregate into insoluble amyloid fibrils that cause myocardial fibrosis, leading to arrhythmias and restrictive cardiomyopathy. The 2 most common types are TTR amyloidosis (ATTR), caused by the liver-synthesized TTR protein, and light chain amyloidosis (AL), caused by monoclonal immunoglobin light chains, a plasma cell dyscrasia. Although a predisposition to ATTR-CM can be inherited as a result of *TTR* gene mutations (variant ATTR-CM) that contribute to TTR protein destabilization and misfolding, ATTR-CM can also be due to spontaneous instability in genetically normal TTR that occurs with advanced age (ATTRwt-CM).

With advances in diagnostic tools and novel therapeutics, ATTRwt-CM is more common than previously appreciated. ATTRwt-CM is present in ≤25% of patients older than 75 years on autopsy^3^ and in ≤16% of patients presenting with heart failure with preserved ejection fraction or aortic stenosis and tendon arthropathies including carpal tunnel syndrome, spinal stenosis, biceps tendon rupture, and the need for joint replacements.^4^ Owing to lack of awareness and multisystem involvement, diagnosis is often delayed for ≤6 years.^5^

Because of the patient’s history of HTx, new graft dysfunction, and medication nonadherence in the setting of dementia, her presentation was concerning for rejection or CAV. ATTRwt-CM was an unanticipated finding on EMBx but was consistent with her advanced age and her history of joint arthropathies.

With advances in immunosuppression, patients are living longer after HTx and are prone to non–HTx-related diseases, including ATTRwt-CM. There is 1 prior case report of newly diagnosed ATTRwt-CM in a 72-year-old patient 15 years after HTx;^6^ however, our case is the first report, to our knowledge, with confirmation of the absence of amyloid from cardiac explant pathologic changes and confirmation of new ATTRwt-CM with mass spectrometry, a proteomics analysis that effectively recognizes all amyloid types in a single assay.^7^

For recipients who undergo HTx for ATTR-CM, the role of amyloid disease-modifying therapies such as tafamidis after HTx is unclear, inasmuch as the slow rate of ATTR deposition may not be clinically relevant when the median post-HTx survival is 12 years.^1^ The potential but untested benefit of tafamidis in halting the progression of extracardiac disease must be weighed against its high cost. However, this case highlights that with improved post-HTx allograft longevity and advancing age, ATTR-CM should be considered in the differential diagnosis of patients who present late after HTx with graft dysfunction, and EMBx may be useful in such patients.

## Follow-Up

After discharge, the patient remained in normal sinus rhythm. A follow-up echocardiogram performed 1 month after hospitalization while the patient was in normal sinus rhythm revealed resolution of left ventricular dysfunction; the ejection fraction was 65%.

## Conclusions

We present an atypical case of an elderly patient with a history of ischemic cardiomyopathy who presented with graft dysfunction 21 years after HTx and with a diagnosis of ATTRwt-CM on EMBx. This case highlights the importance of ATTRwt-CM in the differential diagnosis of patients of advanced age who present decades after HTx with graft dysfunction, especially in the context of other extracardiac manifestations including arthropathies. With advances in immunosuppression and post-HTx care, HTx recipients are living longer and are vulnerable to general diseases of the elderly, expanding the differential diagnosis for graft dysfunction in this population.

## Funding Support and Author Disclosures

The authors have reported that they have no relationships relevant to the contents of this paper to disclose.

## Appendix

Video 1 Transthoracic echocardiogram, apical view with new mildly reduced left ventricular systolic function. **(A)** Apical and **(B)** parasternal long-axis views with new mildly reduced left ventricular systolic function.

Video 2 Transthoracic echocardiogram, parasternal long axis view with new mildly reduced left ventricular systolic function.
